# A dSPACE-based implementation of ANFIS and predictive current control for a single phase boost power factor corrector

**DOI:** 10.1038/s41598-024-63740-2

**Published:** 2024-06-04

**Authors:** Badreddine Babes, Samia Latrèche, Amar Bouafassa, Oualid Aissa, Abd Essalam Badoud, Mabrouk Khemliche, Mohit Bajaj, Ievgen Zaitsev

**Affiliations:** 1 Research Center in Industrial Technologies CRTI, Algiers, Algeria; 2 Automatic Laboratory of Sétif-LAS, Faculty of Technology, University of Ferhat Abbas Setif-1, Setif, Algeria; 3 LGEPC Laboratory, National Polytechnic School of Constantine, Constantine, Algeria; 4 LPMRN Laboratory, Faculty of Sciences and Technology, University of Bordj Bou Arreridj, El Anceur, Algeria; 5 Automatic Laboratory of Sétif-LAS, Faculty of Technology, University of Ferhat Abbas Setif 1, Setif, Algeria; 6 Department of Electrical Engineering, Graphic Era (Deemed to be University), Dehradun, 248002 India; 7 Hourani Center for Applied Scientific Research, Al-Ahliyya Amman University, Amman, Jordan; 8 Graphic Era Hill University, Dehradun, 248002 India; 9 Department of Theoretical Electrical Engineering and Diagnostics of Electrical Equipment, Institute of Electrodynamics, National Academy of Sciences of Ukraine, Peremogy, 56, Kyiv-57, 03680 Ukraine; 10 Center for Information-Analytical and Technical Support of Nuclear Power Facilities Monitoring of the National Academy of Sciences of Ukraine, Akademika Palladina Avenue, 34-A, Kyiv, Ukraine

**Keywords:** Adaptive neuro-fuzzy inference system (ANFIS), Power factor correction (PFC), Total harmonic distortion (THD), Predictive current control (PCC), dSPACE ds1104, IEEE-519 standard, Energy efficiency, Grid stability, Energy science and technology, Engineering, Mathematics and computing

## Abstract

This paper presents an innovative control scheme designed to significantly enhance the power factor of AC/DC boost rectifiers by integrating an adaptive neuro-fuzzy inference system (ANFIS) with predictive current control. The innovative control strategy addresses key challenges in power quality and energy efficiency, demonstrating exceptional performance under diverse operating conditions. Through rigorous simulation, the proposed system achieves precise input current shaping, resulting in a remarkably low total harmonic distortion (THD) of 3.5%, which is well below the IEEE-519 standard threshold of 5%. Moreover, the power factor reaches an outstanding 0.990, indicating highly efficient energy utilization and near-unity power factor operation. To validate the theoretical findings, a 500 W laboratory prototype was implemented using the dSPACE ds1104 digital controller. Steady-state analysis reveals sinusoidal input currents with minimal THD and a power factor approaching unity, thereby enhancing grid stability and energy efficiency. Transient response tests further demonstrate the system’s robustness against load and voltage fluctuations, maintaining output voltage stability within an 18 V overshoot and a 20 V undershoot during load changes, and achieving rapid response times as low as 0.2 s. Comparative evaluations against conventional methods underscore the superiority of the proposed control strategy in terms of both performance and implementation simplicity. By harnessing the strengths of ANFIS-based voltage regulation and predictive current control, this scheme offers a robust solution to power quality issues in AC/DC boost rectifiers, promising substantial energy savings and improved grid stability. The results affirm the potential of the proposed system to set new benchmarks in power factor correction technology.

## Introduction

In the recent years, with the rapidly increasing use of power electronic types of equipment and injection of a huge amount of current harmonics into the distribution net-works, the requirements imposed by international standards and grid codes to their operation as front ends for both distributed energy resources and loads have increased^1,2^. Indeed, switched-mode power supplies (SMPS) with power factor correction (PFC) techniques are mandatory for several industrial applications such as welding, electric vehicles, DC motor drives, and LED lights. Therefore, the PFC circuits are becoming imperative on SMPS as more stringent PQ regulations and strict limits on the total harmonic distortion of input current are imposed such as IEC 61,000–3-2 and IEEE 519^3,4^. Compliance with these standards is crucial for minimizing the adverse effects of harmonics on the electrical grid, such as overheating, equipment malfunction, and reduced efficiency. Active PFC circuits as well as SMPS contain two phases: *i*) a front-end bridge rectifier, and *ii*) a dc-dc converter such as boost, buck-boost, SEPIC, and Cuk for output voltage regulation^5^. PFC boost converters at the front end are required where the preferred output voltage is high. Boost-type circuits are widely employed in commercial power supplies because of their low cost, quite a high efficiency, power factor, and control simplicity^6,7^. The implementation of effective PFC techniques in SMPS has far-reaching implications across various industries. For instance, in the automotive industry, electric vehicles (EVs) rely heavily on efficient power conversion systems to maximize battery life and enhance overall performance^8–10^. Similarly, in industrial automation, precise control of electric drives and welding equipment ensures operational efficiency and product quality. In the context of renewable energy, PFC circuits are crucial for integrating distributed energy resources into the grid, ensuring stability and reliability^11,12^.

One of the major issues in PFC circuits is the development of controller even more robust to load disturbance, and output voltage variations^13^. Literature regarding active power factor correction has introduced many modern control theories focused on achieving high performance against various uncertainties and parameters variations. These control methods were employed to control the output voltage of the AC-DC rectifier, including sliding mode control (SMC)^6,14,15^, fuzzy logic^16,17^, fractional control^18^, and artificial intelligent control^19^. Sliding Mode Control, known for its simplicity and model-free implementation, requires high control efforts, potentially leading to excessive switching, increased losses, and component stress^20–22^. Advanced sliding regulators have been developed to mitigate these issues, but they often involve complex design and implementation processes. Characterized by intuitive and simple concepts, fractional control faces challenges such as computational complexity, real-time implementation difficulties, and sensitivity to interruptions. Techniques such as fuzzy logic controllers (FLC)^23,24^ and artificial neural networks (ANN)^25,26^ manage complex systems without extensive modeling. FLCs handle uncertainties well but require expert knowledge for accurate membership functions (MFs). ANNs complement FLCs by enhancing performance through adaptive mechanisms. The conventional approach of the SMC is commonly utilized in PFC systems. This control technique generally has a simple and model free implementation but quires high control efforts, which may lead to excessive switching and undesirable effects on system components, such as increased losses and component stress^27^. Therefore, to achieve the best result, some sliding regulators have been developed in reference^15^. Where a suitable sliding surface should be determined in order to attain high PF with low THD and fast dynamic reaction. However, there are some limitations, like complex in design and implementation. Fractional control is one of the new advanced control method based on intuitive and simple concepts^18^. Nonetheless, certain constraints exist, such as complexity in computation, difficulty in real time implementation, especially in embedded systems, and tuning, sensitivity to interruptions, and limited robustness^18^.

Artificial control schemes like fuzzy logic controller and artificial neural networks have been intensively applied in several industrial applications because they are not subjected to systems modeling. A fuzzy logic controller is broadly used for controlling complex systems with uncertainties; it does not demand the understanding of the mathematical background and the complicated mechanisms of the studied system. However, the fuzzy system needs the knowledge of an expert, which is usually expressed in linguistic terms and by accurate membership functions (MFs). For these reasons, artificial neural networks based on learning ability can make up for this lack and enhance the performance of fuzzy logic. By fusing the human-like thinking ability of the FLC system and the learning capability of the artificial neural network, an adaptive neural-fuzzy inference system (ANFIS) is designed, which generates results more accurately regarding to other schemes that used only fuzzy system or neural network^28–31^. Moreover, the ANFIS offers more computing advantages by eliminating the construction of mathematical models, thereby decreasing the computational time needed and maintaining consistent performance in the presence of perturbations^19^. The ANFIS approach is implemented in this paper to enhance the performance of a single-phase PFC rectifier; hence, it ensures a controlled dc-link voltage regardless of the dynamic parameters' changes such as load and reference output voltage. Moreover, the ANFIS controller is used to generate the peak value of the reference current for the inner current loop^32,33^.

Among the advanced control strategies that have been widely applied in recent years for the PQ enhancement of the single-phase active PFC rectifier, we will cite the predictive control method. This control technique is based on a very simple concept. It achieves unity power factor by controlling the inductor current to be in phase with the input rectified voltage^34^. It has been the standard strategy for single-phase PFC rectifier in the industry because it has the advantage of lower THD, has improving noise and easily forming source current waveforms. So, to choose an efficient input current control scheme, predictive current control is an appropriate option because it offers some fast transient responses, better robustness, and low implementation complexity. There are many works in the literature focusing on predictive current control^6,17,35^. A cascaded model-free predictive control approach based on the unified ultra-local model is proposed in reference^36^ for single-phase boost power factor correctors are presented to increase control performance and resilience. With a set switching frequency and dead-time compensation, a model predictive current control is presented to lower the grid-side current's total harmonic distortion (THD) in reference^37^. For four-level hybrid-clamped converters, the authors of reference^38^ proposed a straightforward model predictive control system that achieves high-quality current control and robust voltage balancing. It is demonstrated that predictive current control can have improved performance rates at different applications. By adapting predictive control strategies for multi-phase systems and integrating them into larger power grids, it becomes possible to address a wide range of challenges, including voltage balancing, fault detection, grid stability, demand response management, and energy trading optimization^39,40^. These control strategies not only improve the reliability and efficiency of energy systems, but also contribute to the integration of renewable energy sources, grid modernization, and sustainable energy transition efforts.

Accordingly, in this work, the predictive current control is proposed to wave shaping of input current and enhances the PF, which is missing in the literature. The literature underscores persistent challenges in power factor correction (PFC) strategies for AC/DC boost rectifiers, necessitating innovative approaches to enhance energy efficiency and grid stability. Conventional rectification methods often yield distorted input currents and low power factors, posing challenges in meeting regulatory standards and optimizing energy utilization. Additionally, dynamic operating conditions, such as load variations and input voltage fluctuations, further exacerbate these issues, highlighting the need for robust and adaptive control schemes. Existing control approaches have demonstrated limited effectiveness in achieving the desired power quality targets under varying operating conditions. Traditional proportional-integral-derivative (PID) controllers and linear control techniques struggle to adapt to nonlinearities inherent in AC/DC boost rectifiers, leading to suboptimal performance and compromised power factor correction. Moreover, the complexity of designing control algorithms capable of addressing dynamic system behavior and uncertainties remains a significant challenge.

To cover the gap in the literature, the design, simulation, and hardware implementation of single-phase active PFC based on DC bus voltage loop ANFIS controller and predictive current controller have been performed in this paper. The suggested predictive ANFIS control scheme for the single-phase active PFC is tested in simulation and con-firmed in real-time using dSPACE DS1104 card. The simulation and experimental results have been responded to the power quality requirements and follow-up of reference output voltage regardless of output voltage variations and load changes, which allows realizing high power factor for the power grid and low THD. The benefits of the suggested control scheme are:

- Less complicated architecture of the proposed control strategy;
- Fast responses;
- High dynamic performance and robustness under the step type variations in the output voltage and in the load;
- Small fluctuation and low overshoots for different operating conditions.

To this end, this paper suggests a new cascade control for single-phase active PFC boost converter with an ANFIS controller in a voltage loop and performing current control with a predictive controller. In addition, this work presents the control system design and its implementation around dSPACE 1104 card. The main contributions are summarized as follows:

1. Improving the performance of PFC such as PF, THD, and output voltage regulation.
2. A holistic control strategy is synthesized for current and voltage loops of a single-phase PFC rectifier using a robust cascaded ANFIS-Predictive controller, which is a new effective controller with a fixed-sampling frequency that has not been applied before for PFC control design.
3. Both the abovementioned theoretical analysis and the proposed cascaded ANFIS-Predictive controller of the single-phase PFC rectifier are validated by simulation and experimental results.

The remainder of this paper is arranged as follows: Section "Modeling of PFC boost converter" presents the modelling of the PFC circuit. In Section "Proposed control design", the study of proposed controllers is mentioned. Simulation results and diverse test cases including change in load and in output voltage are granted in Section "Simulation results and discussion". The proposed controller is tested in real-time by using the dSPACE card in Section "Identified practical challenges". Final remarks can be found in Section "Experimental validation".

## Modeling of PFC boost converter

**Figure 1:**
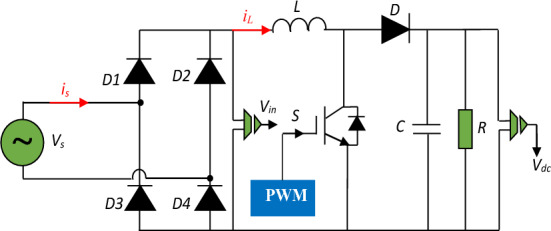
A-PFC boost converter structure.

The dc-dc boost converter is employed in the present paper for achieving the best operations of the system regardless of the load changes with low cost-effective. The basic structure of the APFC pre-regulator is shown in Fig. 1. The power circuit involves of an uncontrolled full-bridge rectifier followed by a boost converter. When the IGBT is switched ON, the output capacitor supply energy to load and the inductor stores the energy^5,41^. When switch is turns OFF, the inductor charges the output capacitor and gives energy for the output load. Taking into consideration the two operation modes, the average model of the circuit is given by the following equation^15^:

1 $$\left\{\begin{array}{c}C\frac{d{v}_{dc}}{dt}=\left(1-S\right){i}_{L}-\frac{{v}_{dc}}{R}\\ L\frac{d{i}_{L}}{dt}={v}_{in}-\left(1-S\right){v}_{dc}\end{array}\right.$$

where the *v*_*in*_ is the input voltage, *v*_*dc*_ is the output voltage of the circuit; *i*_*L*_ is the input inductance current and *S* is the switching state.

To get a sinusoidal input current (*i*_*s*_), the input voltage sees a resistive load equal to the ratio of *v*_*in*_ and *i*_*L*_. The procedure has been accomplished as follows; the supply voltage is sensed, and its unit template is generated by using a PLL circuit that acts as a sinusoidal reference for the input current ^42^. The reference input current (*i*_*ref*_) is produced as the product of the outer voltage loop and the unit template of the PLL. In this way, the input current (*i*_*s*_) is enforced to follow the sinusoidal shape of a PLL to attain unity PF ^17^.

## Proposed control design

**Figure 2:**
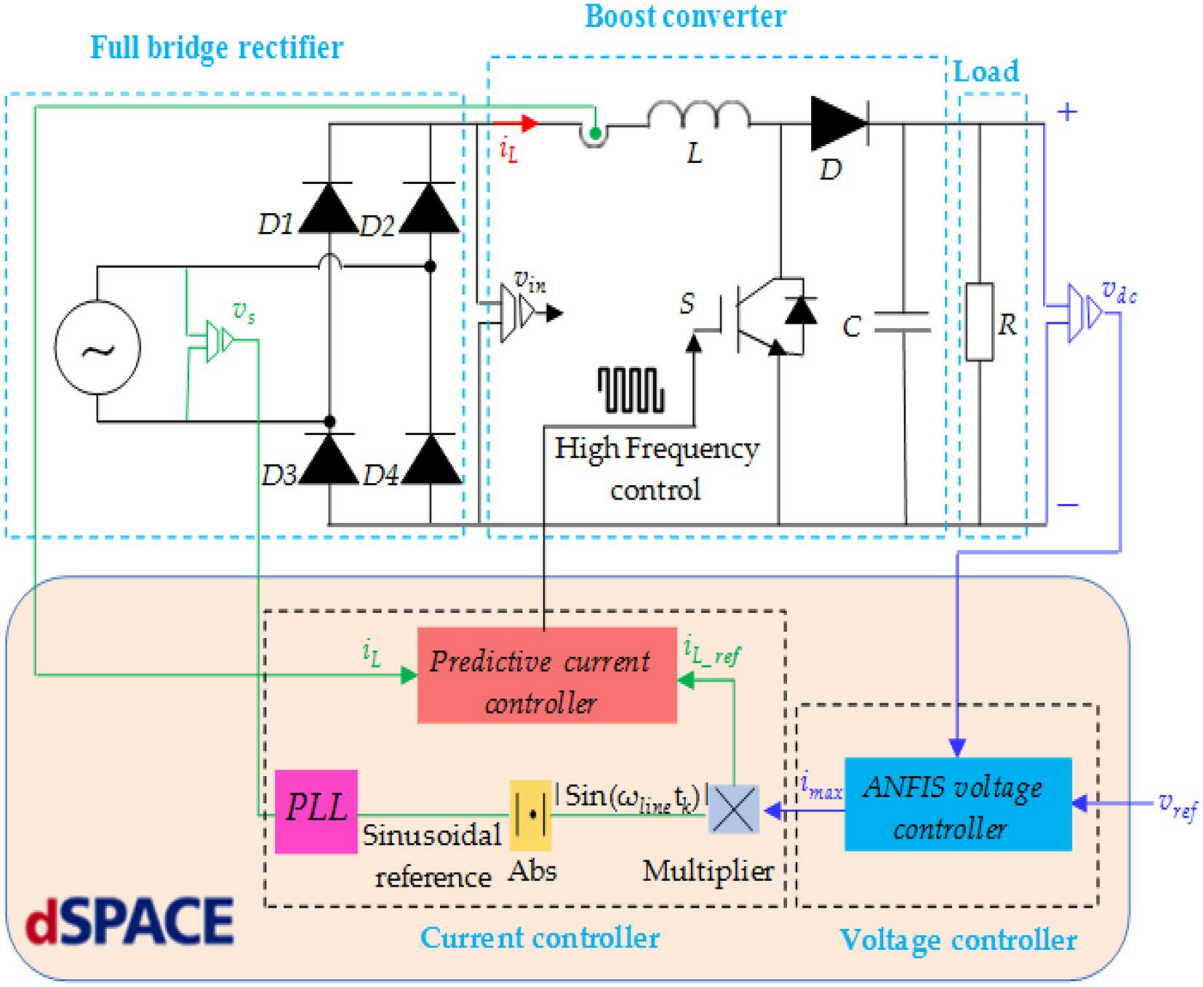
Schematic diagram of APFC converter with control loops.

The proposed controller of the active PFC rectifier includes a dc-link output voltage and input current dual control loop. A description of the studied system with the suggested control algorithms is depicted in Fig. 2. The studied system involves of AC grid supplying a dc-dc converter through the uncontrolled full bridge. The system under study experiences several control challenges because of uncertain-ties from both the dc-link voltage and load. The unexpected load brings up instability and power quality issues of the system if they do not be controlled carefully by the controller unit. Indeed, the system under study contains two control loops; the ANFIS based dc-link voltage and predictive current controllers. The ANFIS controller is applied to control the output voltage of the boost converter, whose inputs are: (*i*) the error (*e*) between the measured output voltage and its reference (*e* = *v*_*dc*_ – *v*_*ref*_), and (ii) the error change (*de*), whereas its output is the peak value of reference inductor current. The second controller is the predictive control, which use to wave shaping of supply current and improve the power quality of the circuit.

### ANFIS based DC-link voltage controller

Jang developed the adaptive neuro-fuzzy inference system (ANFIS)^43–45^. ANFIS integrates the robust learning capabilities of neural networks with the intuitive reasoning approach of fuzzy logic. This hybrid structure allows ANFIS to handle nonlinearities and uncertainties within control systems more effectively than traditional methods. In an ANFIS system, the membership functions (MFs) and fuzzy rules, which form the core of FLC, are dynamically adjusted and optimized using the learning algorithms of ANN. This integration enhances the system's adaptability and precision in controlling complex processes. The key to ANFIS's effectiveness lies in its ability to fine-tune the fuzzy inference system (FIS) parameters. The ANN component of ANFIS is responsible for adjusting the MFs and rules based on input–output data, thereby optimizing the control parameters to minimize errors. This continuous learning process ensures that the ANFIS controller remains effective under varying conditions and disturbances. ANFIS employs a hybrid learning algorithm that combines gradient descent and least squares estimation. This algorithm iteratively updates the parameters of the MFs to reduce the difference between the actual output and the desired output. By utilizing both supervised learning (from provided datasets) and unsupervised learning (from system feedback), ANFIS can achieve an optimal mapping between inputs and outputs, leading to improved control performance.

**Figure 3:**
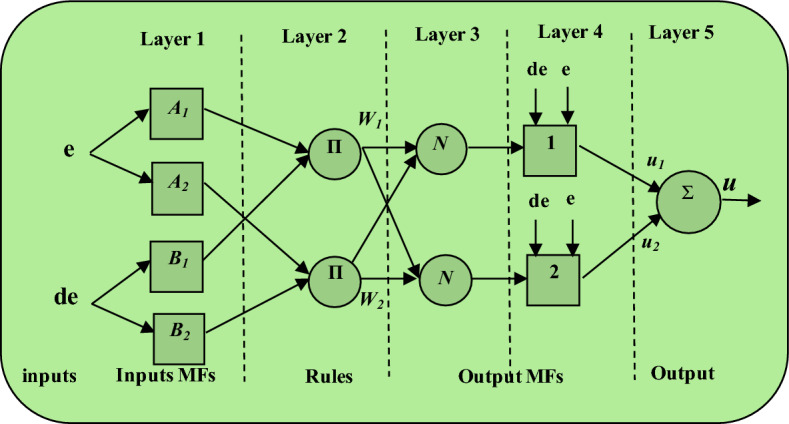
Typical five layers of ANFIS.

The typical architecture of ANFIS is depicted in Fig. 3. This architecture comprises five distinct layers, each with a specific role in the signal processing and decision-making process.

Fuzzification Layer (Layer 1) converts crisp input values into fuzzy sets using predefined membership functions. Each node in this layer represents a fuzzy rule, which can be adjusted adaptively. Nodes in Rule Layer (Layer 2) layer represent the antecedent part of the fuzzy rules. The outputs of this layer are the firing strengths of the rules, calculated as the product of the membership values from Layer 1. Normalization Layer (Layer 3) normalizes the firing strengths of the rules by dividing each firing strength by the sum of all firing strengths. In Defuzzification Layer (Layer 4), the normalized firing strengths are used to compute the weighted output values based on the consequent parameters of the fuzzy rules. Output Layer (Layer 5) aggregates the weighted outputs from Layer 4 to produce a single crisp output.

In the architectural diagram (Fig. 3), each node performs specific operations on the incoming signals. Adaptive nodes, indicated by squares, adjust their parameters through the learning process, while fixed nodes, represented by circles, perform predetermined operations without modification. By combining the human-like reasoning capabilities of FLC with the adaptive learning features of ANN, ANFIS can effectively manage control systems with complex dynamics and uncertainties. This makes it particularly suitable for applications such as power factor correction (PFC) in single-phase rectifiers, where maintaining stable operation under varying loads and voltage conditions is crucial. In summary, ANFIS enhances the performance and reliability of control systems by dynamically adjusting to changing conditions and minimizing control errors, thereby providing a robust solution for modern control challenges.

**Figure 4:**
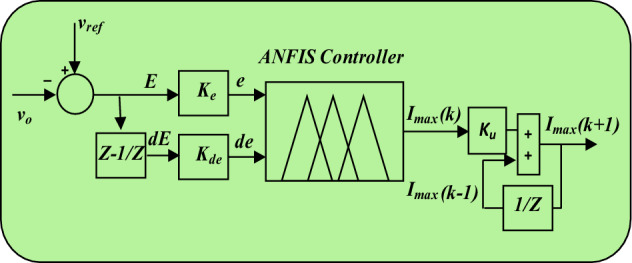
NFIS controller.

The ANFIS has two inputs (Fig. 4)^46^, the first is the error (*e*) between the sensed output voltage and its reference and the second input consists of the variation in voltage error (*de*). The output is the peak value of the reference current (*I*_*max*_).

According to Fig. 4, the ANFIS controller is normalized by using scaling factors (*K*_*e*_, *k*_*de*_ and *k*_*u*_) to get an optimal performance as fallow:

2 $$\left\{\begin{array}{c}{I}_{max}(k+1)={I}_{max}(k-1)+{K}_{u}.{I}_{max}(k)\\ e(k)={K}_{e}.E(k)\\ de(k)={K}_{de}.dE(k)\end{array}\right.$$

The if–then rules of the Sugeno fuzzy system are given as follows ^19^:

$$\begin{array}{c}Rule 1:if\, e\, is\, {A}_{1} \,and\, de\, is\, {B}_{1}, then\, {u}_{1}={p}_{1}e+{q}_{1}de+{r}_{1} \\ Rule 2:if\, e\, is\, {A}_{2}\, and\, de\, is\, {B}_{2}, then\, {u}_{2}={p}_{2}e+{q}_{2}de+{r}_{2}\end{array}$$

where: (*e*) and (*de*) are the inputs to ANFIS; *A*_*i*_ and *B*_*i*_ are the antecedent membership functions; *u*_*i*_ is the output; *p*_*i*_ and *q*_*i*_ represent the weighting factors for *ith* input and *r*_*i*_ is the *ith* output bias. From Fig. 3, the ANFIS consists of five layers; the main goal of each one is given as follows:

Layer 1 (fuzzification): neurons in this layer perform fuzzification of crisp inputs. Each node is an adaptive node; the number of these nodes equals the number of variables. This layer can be modelled by the following equation:

3 $${O}_{i}^{1}={\mu }_{A1}\left(E\right)=\frac{1}{1+{\left({\left(\left(E-{c}_{1}\right)/{a}_{1}\right)}^{2}\right)}^{{b}_{1}}}$$

Layer 2 (rule): every node in this layer is a fixed node labelled as π represents a premise of a rule. The output of this layer is a product of the incoming signals by the T-norm product operator as follows:

4 $${{O}_{i}^{2}=w}_{i}={\mu }_{A1}\left(e\right)\times {\mu }_{B1}\left(de\right) $$

where (*w*_*i*_) is the firing strength of rule *i*, (*e*) and (*de*) are the input error and its change variables respectively.

Layer 3 (normalization): this layer normalizes the firing strength of each rule according to the following equation:

5 $${O}_{i}^{3}={\overline{w}}_{i}=\frac{{w}_{i}}{{w}_{1}+{w}_{2}} , (i=\text{1,2})$$

Layer 4 (defuzzification): The defuzzification neuron calculates the weighted consequent value by the following relation:

6 $${O}_{i}^{4}={\overline{w}}_{i}\times {u}_{i}={\overline{w}}_{i}\times \left({p}_{1}E+{q}_{1}DE+{r}_{1}\right)$$

where: *p*_*i*_, *q*_*i*_, and *r*_*i*_ are the consequent parameters.

Layer 5 (output): it is the output layer; it computes the overall output value which is linear in consequent parameters by summing all the incoming signals as per:

7 $${O}_{i}^{5}=u={\sum }_{i}{\overline{w}}_{i}\times {u}_{i}$$

Input variables characterizing the ANFIS controller are the *e*, *de* and its output (the peak value of the reference current *I*_*max*_). The training of ANFIS has been done using the back-propagation algorithm. The whole process of ANFIS involves four steps: (*i*) loading the training data from the model which need to be learned; (*ii*) converting the training data into a fuzzy set by designating a suitable MFs and creating the “IF–THEN” rules; (*iii*) updating the parameters based on the training algorithm; (iv) converting the fuzzy values to numerical values and saves the given model.

The FLC is employed to train the proposed ANFIS scheme, where it is implemented on the dSPACE ds1104 control board^47^. After that, the inputs and output values are employed for ANFIS controller training. The number MFs of the inputs *e* and *de* and output are selected based on the trial–error procedure. Seven linguistic variables are created for implementing the ANFIS controller such as; Negative Big (NB), Negative Mean (NM), Negative Small (NS), Zero (Z), Positive Small (PS), Positive Mean (PM), and Positive Big (PB).

**Figure 5:**
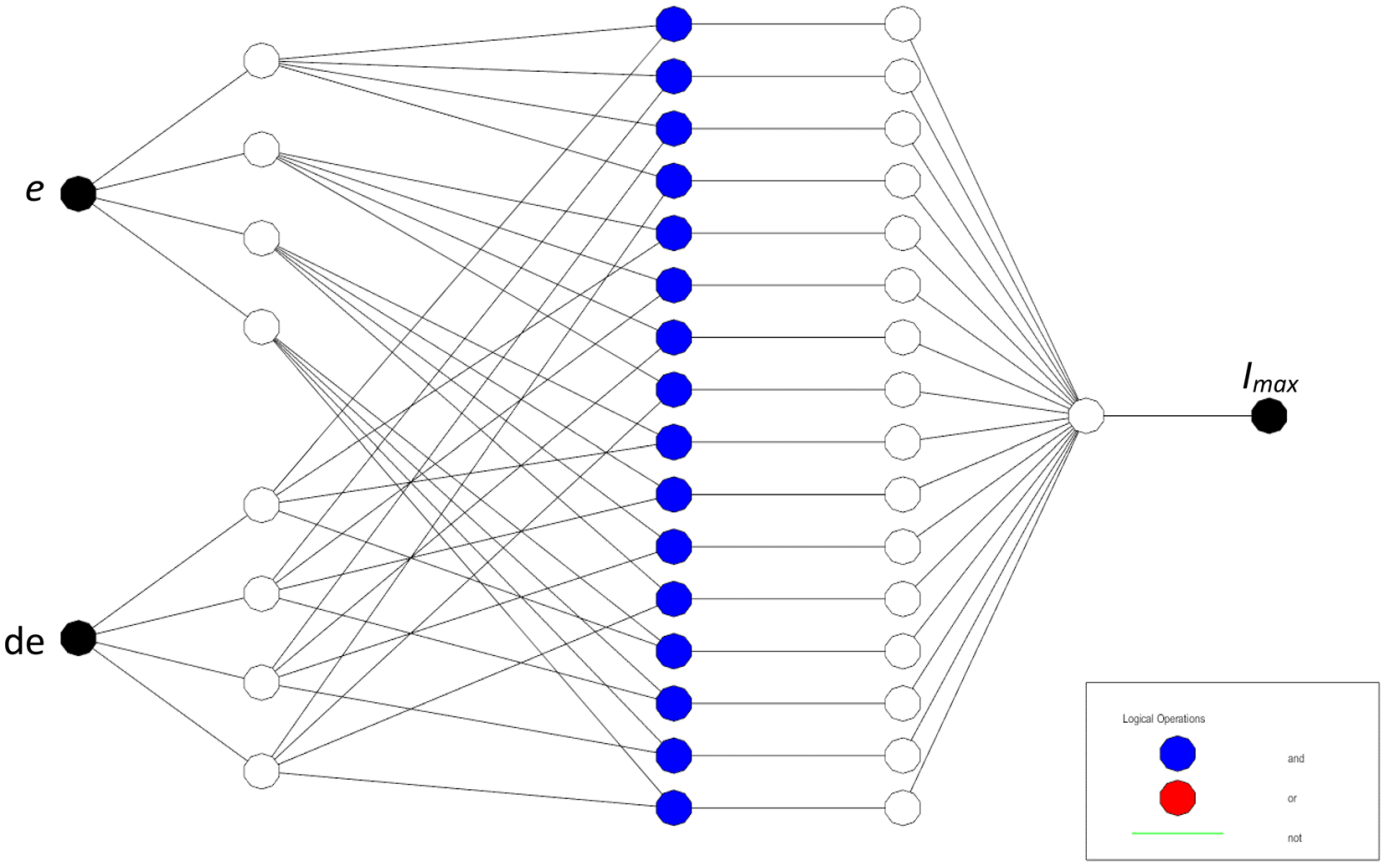
ANFIS structure.

**Figure 6:**
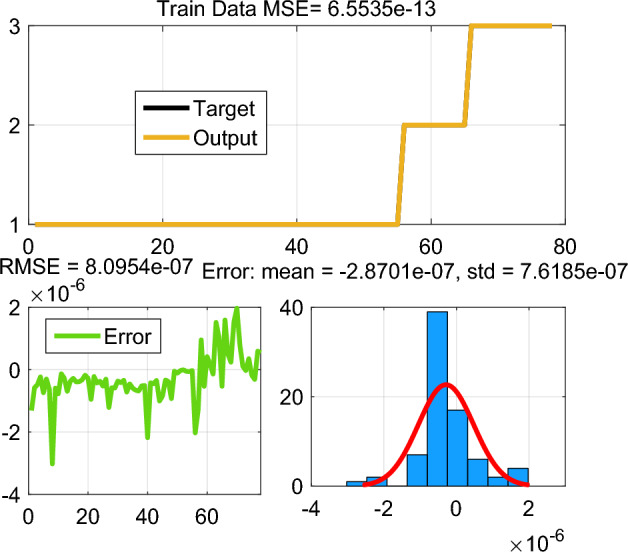
Train error with hybrid learning algorithm.

**Figure 7:**
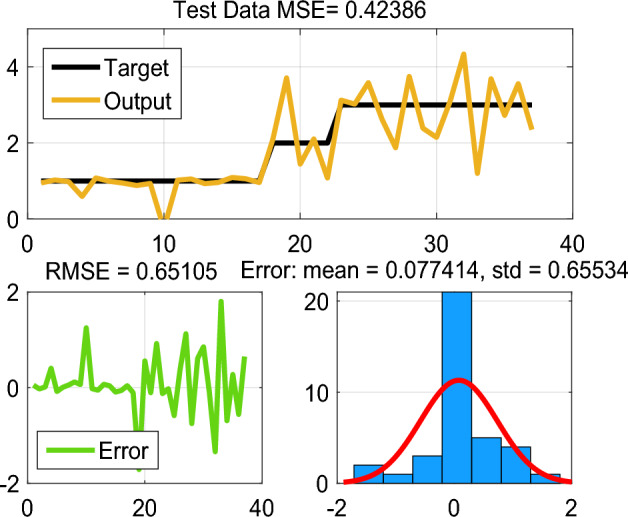
Test error with hybrid learning algorithm.

**Figure 8:**
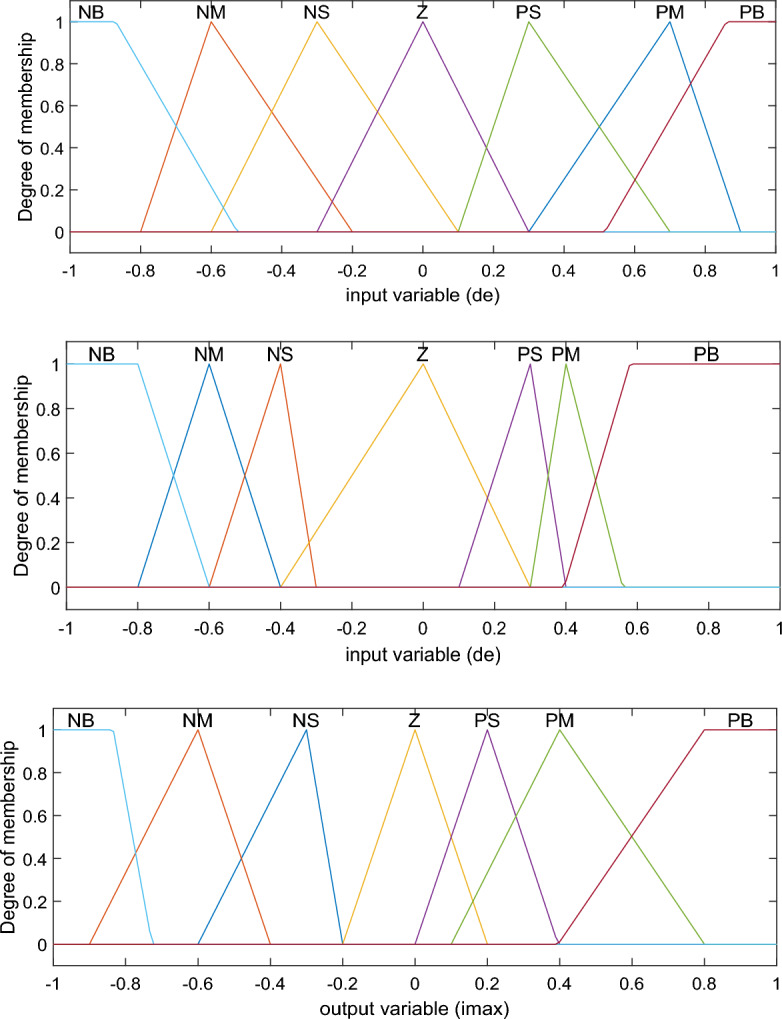
Inputs and output variables defined in the developed ANFIS.

This paper enhances the DC-link voltage regulation by investing in the ANFIS scheme. The fuzzy logic-based controller of DC-link voltage regulation is used and its data are used for ANFIS parameters training. The MF parameters are fine-tuned through a learning process employed the hybrid algorithm, which is commonly used in training neural networks. The ANFIS architecture includes several layers of the ANN structure, as shown in Fig. 5. The network model of ANFIS regulator is trained, tested, and validated using 115 data sets in total using MATLAB v. R2023a Fuzzy logic toolbox. Out of the 78 data sets, were used to train the ANFIS regulator, and 37 were utilised for validation. The summary parameters of the ANFIS model is presented in Table 1. Figures 6 and 7 show how well the ANFIS regulator learned the training data and its ability to estimate the maximum current with high precision. The used rules of the developed ANFIS controller are tabulated in Table 2. Figure 8 displays the MFs of the Sugeno-type fuzzy inference systems after training.

**Table 1:** Rule table of ANFIS regulator used in the PFC system.

Parameters	Types/values
Fuzzy structure	Takagi–Sugeno
Input layer	2
Output layer	1
Input MF number	[7 7]
Rules	49
Optimization method	Hybrid algorithm
Training epochs	200

**Table 2:** Rule table of ANFIS used in the PFC system.

Fuzzy sets of output I_max_(k)		*de(k)*						
		NB	NM	NS	Z	PS	PM	PB
*e(k)*	NB	NB	NB	NB	NB	NM	NB	Z
	NM	NB	NB	NB	NM	NS	Z	PS
	NS	NB	NB	NM	NS	Z	PS	PM
	Z	NB	NB	NS	Z	PS	PM	PB
	PS	NM	NB	Z	PS	PM	PB	PB
	PM	NS	Z	PS	PM	PB	PB	PB
	PB	Z	PS	PM	PB	PB	PB	PB

**Figure 9:**
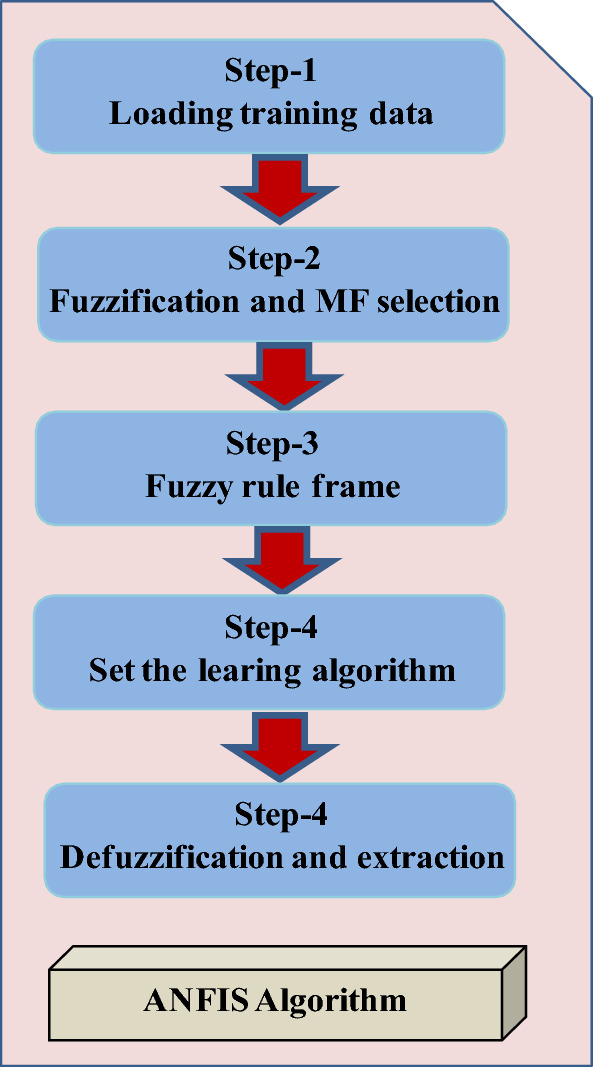
ANFIS flowchart.

After that, the ANFIS controller has taken place to get better performances and robustness during the changes in the parameters of the studied PFC system. The flowchart of the suggested ANFIS is presented in Fig. 9, which comprises 04 steps; the first-step is loading the training data. Then, the training data converted into fuzzy set. The “if–then” rules will be created according to weighted fuzzified input. Then, a training method based on minimisation of the error has been used to update the parameters of the controller. Finally, the defuzzification process transforms the fuzzified data to a numerical data and saves the obtained model.

### Inner current control loop

**Figure 10:**
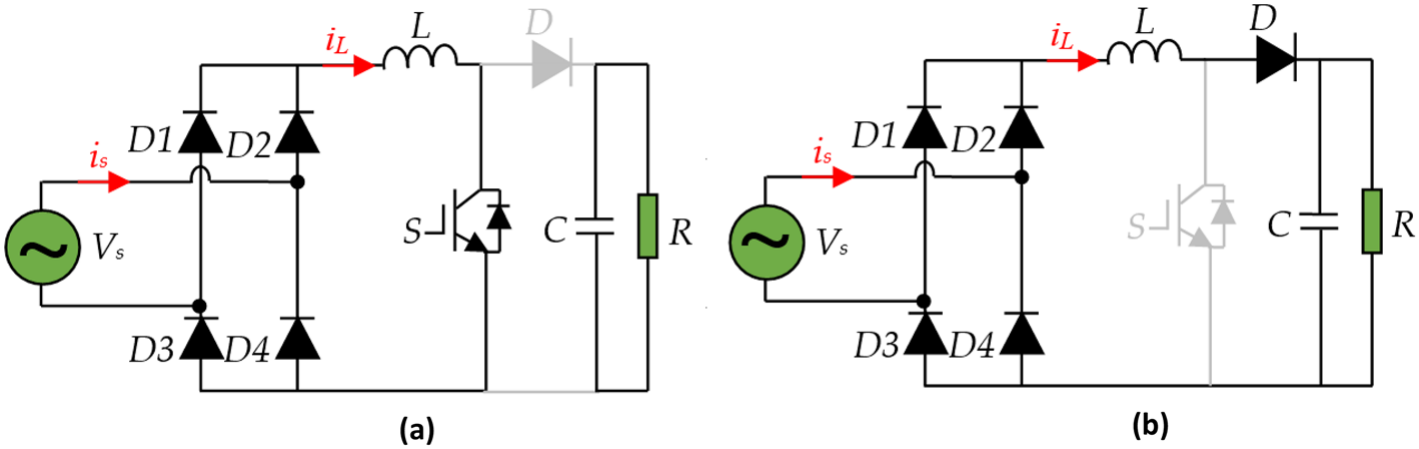
Operation modes of PFC Boost converter: (a) when the switch is ON, and (b) when the switch is OFF.

In this loop, primary emphasis is placed on minimizing the error between the inductor current and its reference. Among several techniques proposed for inductor current control, the predictive technique is one of the most significant candidates that have given extremely promising results during laboratory testing. The use of predictive current control is a very interesting option because of its simplicity and low cost-effective technique. Moreover, the predictive current control (PCC) allows controlling several industrial systems taking into account nonlinearities and uncertainties, which greatly reduces the controller complexity^48,49^. In this paper, the PCC is implemented to ensure a unity power factor and low THD by controlling the input current regardless of the changes in the treated system parameters. The PFC boost circuit has two configurations, one when the switch is ON and the other when it is OFF as shown in Fig. 10.

Considering the switch states, the inductor current *i*_*L*_(*t*) when the switch *S* is turned ON or turned OFF can be written as:

- S turned ON:

8 $$\text{L}\frac{{\text{di}}_{\text{L}}}{\text{dt}}={\text{v}}_{\text{in}}\left(\text{t}\right);\text{ t}\left(\text{k}\right)\le \text{t}\le \text{t}\left(\text{k}\right)+\text{d}\left(\text{k}\right).{\text{T}}_{\text{s}}$$

- S turned OFF:

9 $$\text{L}\frac{{\text{di}}_{\text{L}}}{\text{dt}}={\text{v}}_{\text{in}}\left(\text{t}\right)-{\text{v}}_{\text{dc}}\left(\text{t}\right);\text{ t}\left(\text{k}\right)+\text{d}\left(\text{k}\right).{\text{T}}_{\text{s}}\le \text{t}\le \text{t}\left(\text{k}+1\right)$$

**Figure 11:**
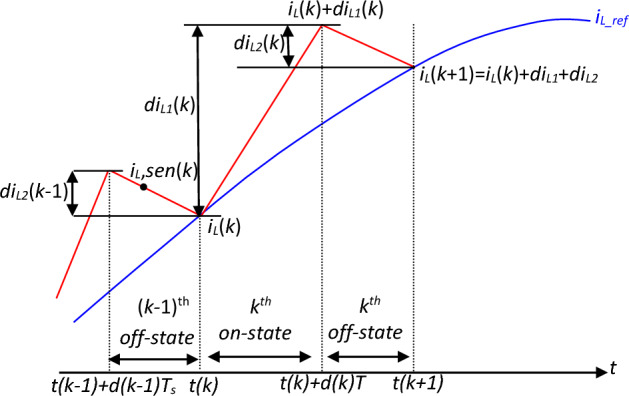
Inductor current waveform in one period.

The inductor current *i*_*L*_(*t*) for one switching cycle is depicted in Fig. 11.

From Fig. 11, the inductor current can be expressed as:

At: *t*(*k*) + *d*(*k*).*T*_*s*_

10 $${i}_{L}\left(t\left(k\right)+d\left(k\right).{T}_{s}\right)={i}_{L}\left(t\left(k\right)\right)+\frac{1}{L}{v}_{in}\left(t\left(k\right)\right).d\left(k\right).{T}_{s} $$

At: (k + 1)th

11 $${i}_{L}\left(t\left(k+1\right)\right)={i}_{L}\left(t\left(k\right)+d\left(k\right).{T}_{s}\right)+\frac{1}{L}\left({v}_{in}\left(t\left(k\right)\right)-{v}_{dc}\left(t\left(k\right)\right).\left(1-d\left(k\right)\right)\right){.T}_{s} $$

From Eqs. (10 and 11), the discrete form of inductor current can be written as:

12 $${i}_{L}\left(k+1\right)={i}_{L}\left(k\right)+\frac{{T}_{s}}{L}\left({v}_{in}\left(k\right)-{v}_{dc}\left(k\right).\left(1-d\left(k\right)\right)\right) $$

From Eq. (12), the duty cycle can be expressed as:

13 $$d\left(k\right)=\frac{L}{Ts}\frac{{{i}_{L}\left(k+1\right)-i}_{L}\left(k\right)}{{v}_{dc}}+\frac{{v}_{in}\left(k\right)-{v}_{0}\left(k\right)}{{v}_{dc}} $$

Substituting *v*_*dc*_(*k*) and *i*_*L*_(*k* + 1) in Eq. (13) by its references, the duty cycle can be calculated as:

14 $$d\left(k\right)=\frac{L}{Ts}\frac{{{i}_{L\_ref}\left(k+1\right)-i}_{L}\left(k\right)}{{v}_{ref}}+\frac{{v}_{in}\left(k\right)-{v}_{ref}\left(k\right)}{{v}_{ref}} $$

where the predicted reference current *i*_*L_ref*_ (*k* + 1) is calculated as per Eq. (15):

15 $${i}_{L\_ref}\left(k+1\right)={I}_{max}.\left|sin ({\omega }_{line}.t\left(k+1\right)\right| $$

where *I*_*max*_ is given by the ANFIS controller.

**Figure 12:**
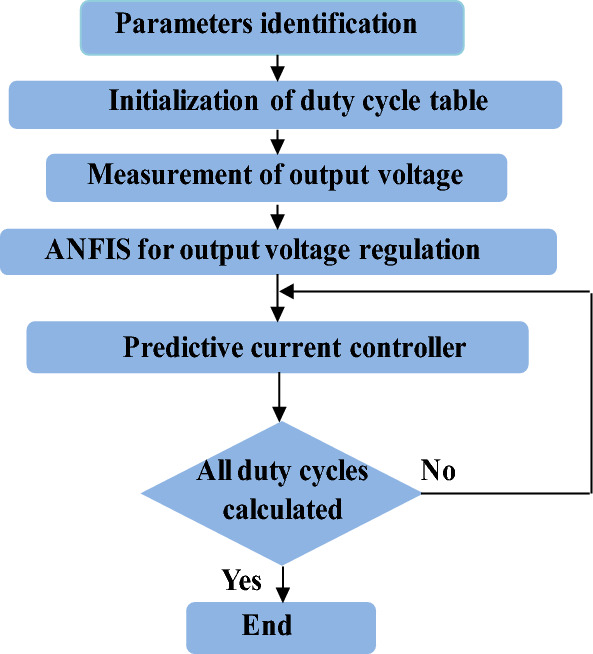
Flowchart of proposed method.

All steps of the suggested control scheme are presented in Fig. 12.

- *Step 1* Identification of the constant parameters (inductance value, switching frequency…etc.).
- *Step 2* Determinate of reference output voltage and establish the ANFIS for output voltage regulation.
- *Step 3* Determinate *i*_*L_ref*_ through ANFIS with frequency equal to input line frequency (*ω*_*line*_) at the present instant and introduce the predictive current control.
- *Step 4* Verified that all duty cycles are measured by the predictive current controller.

## Simulation results and discussion

The following assumptions were made before analyzing the performances of the proposed control approach:

- CCM operation is assumed;
- Semiconductor elements are ideal;
- Unity PF is assumed, the source current is in phase and shape with the input voltage.
- PFC output voltage is DC with negligible ripple.
- Inductor current ripple is assumed to be half of peak inductor current.

To evaluate the performance and effectiveness of the proposed control strategy, the system under investigation was modeled and simulated in the MATLAB/Simulink environment for different operating cases. The most important parameters of the studied PFC system are given in Table 3.

**Table 3:** Specific parameters of PFC system.

Components	Values	Unit
AC supply voltage (RMS)	70	V
Output capacitor	1100	μF
Load resistor	50–100	Ω
Switching frequency	20	kHz
Output reference voltage	180–220-250	V
Input inductor	20	mH
Sampling period	50	μs

### Steady-state performance

**Figure 13:**
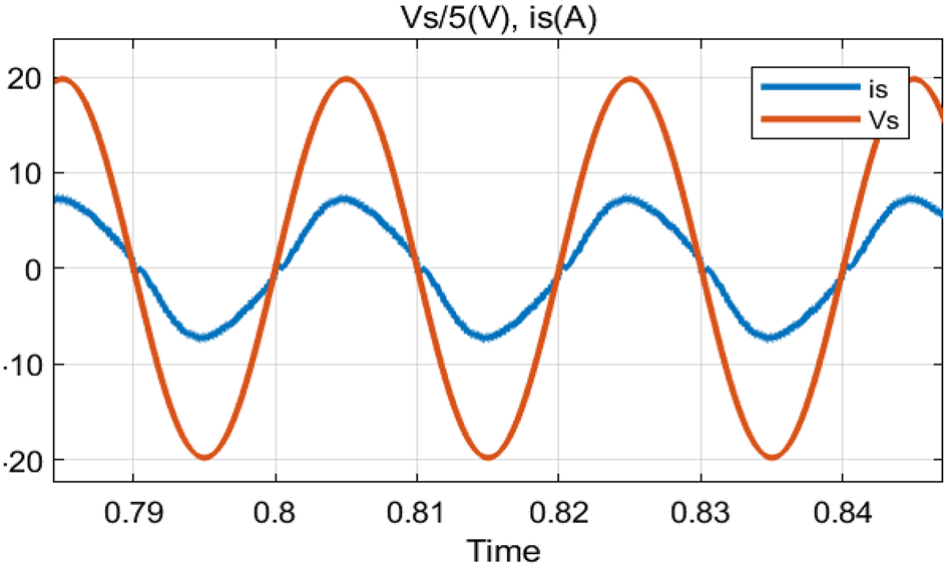
Input voltage (*v*_*s*_/5) response and input current (*i*_*s*_) response.

**Figure 14:**
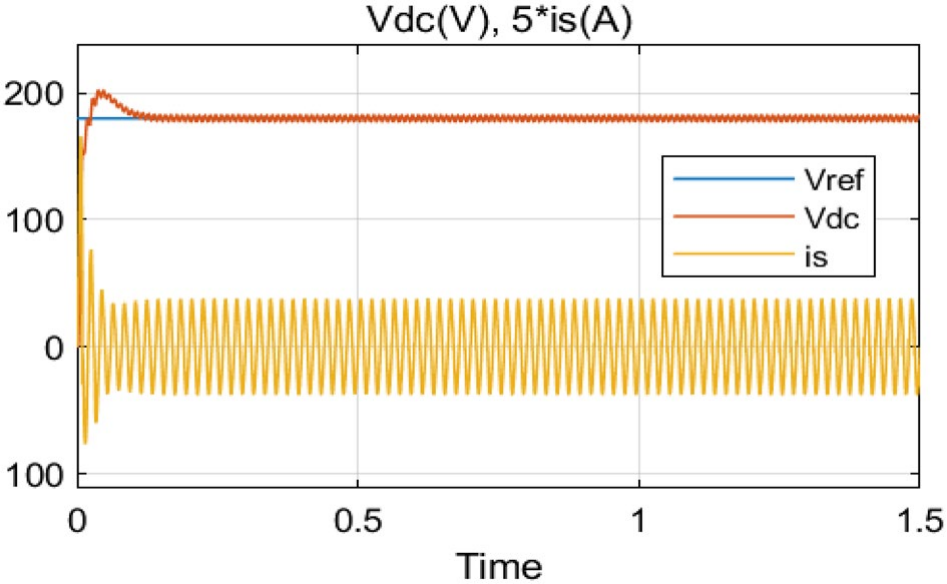
Output voltage (*v*_*dc*_) response and input current (5*i_*s*_) response.

**Figure 15:**
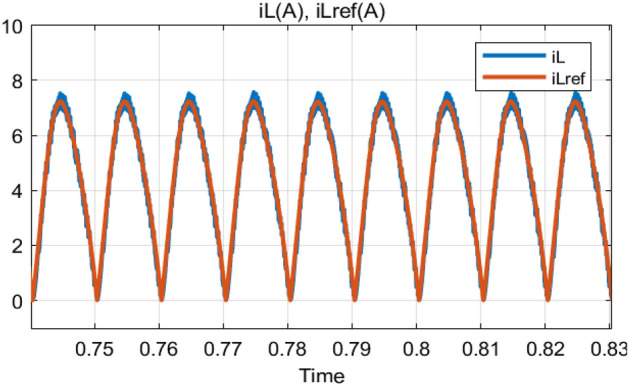
Inductor current (*i*_*L*_) response and its reference current (*i*_*L_ref*_).

**Figure 16:**
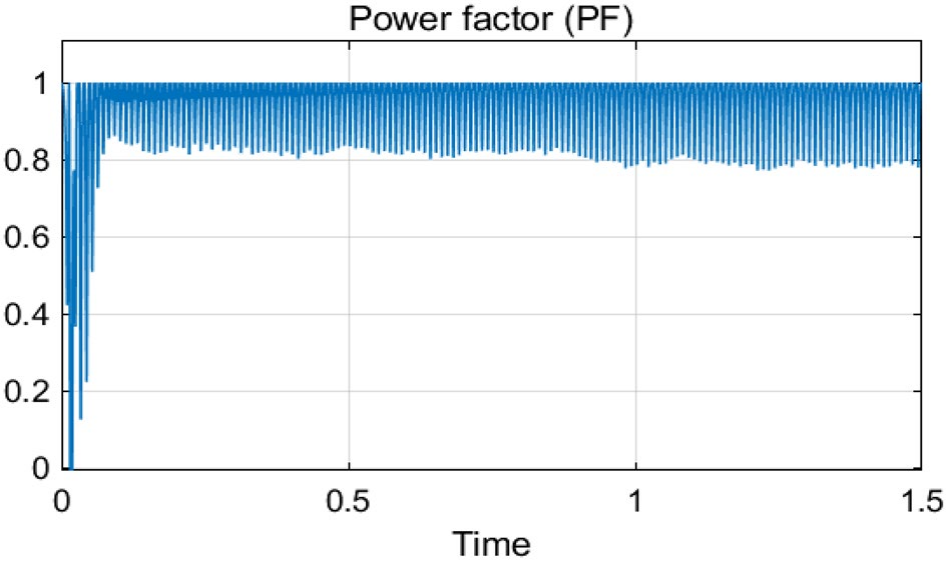
Measured PF at AC grid.

The Steady-State performance of AC-DC PFC boost converter based on ANFIS and PCC with fixed DC-load (*R* = 100 Ω) and output voltage (*v*_*ref*_ = 180 V) are shown in Figs. 13, 14, 15, 16. Thanks to the proposed control, the following comments are deduced from the obtained simulation results:

1. Perfect input current shaping as shown in Fig. 13.
2. The output voltage (*v*_*dc*_) tracks its desired value perfectly with a fast response time (less than 100 ms) as illustrated in Fig. 14.
3. The inductor current (*i*_*L*_) tracks its reference as depicted in Fig. 15.
4. Highly power factor as seen in Fig. 16.

### Transient performances

**Figure 17:**
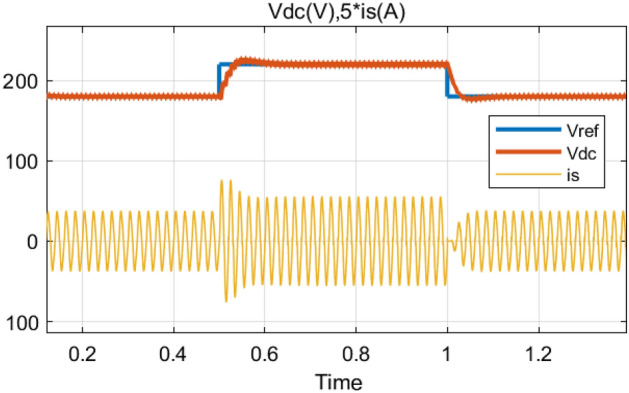
Simulation results of reference output voltage variations from *v*_*dc*_ = 180V to *v*_*dc*_ = 220 V, and inversely.

**Figure 18:**
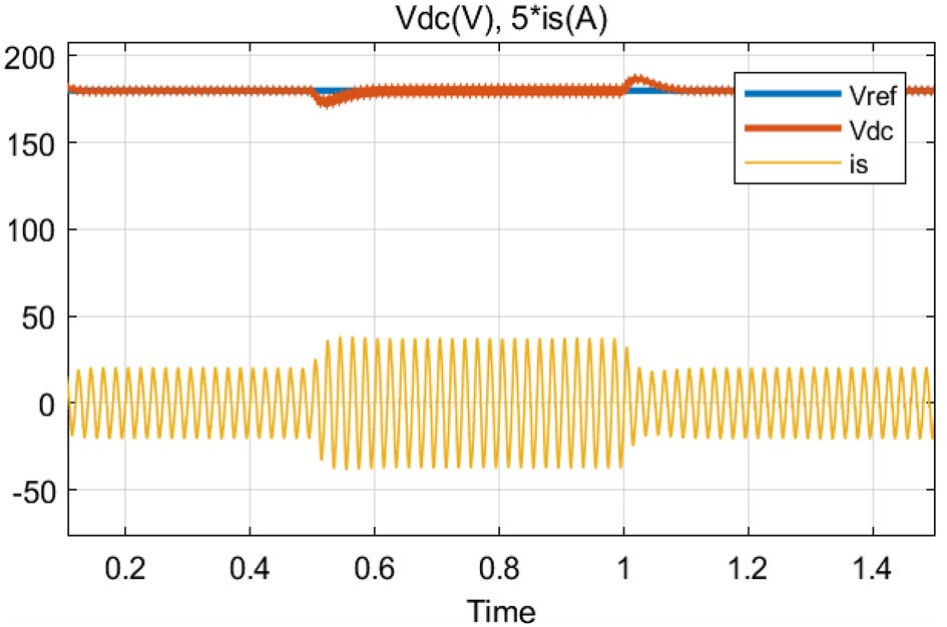
Simulation results of a step-change of the load from 100 to 50 Ω and inversely.

In order to examine the applied control performance during a transient mode; (*i*) the reference output voltage is increased from 180 to 220 V and inversely with a fixed load resistor at 100 Ω (Fig. 17). Considering Fig. 17, it can be clearly seen the output volt-age (*v*_*dc*_) tracks its reference perfectly with a fast response time (less than 100 ms) and low overshoot. Also, the input current rest purely sinusoidal which leads to achieving a high PF. (*ii*) a 50% step decrease and 50% step increase in the load is applied at time instant *t* = 0.5 s and *t* = 1s, respectively, with a fixed output voltage of 180 V (Fig. 18). From Fig. 18, it is clear that the output voltage (*v*_*dc*_) remains stable at desired value with a small fluctuation.

### Robustness to parameter variations

**Figure 19:**
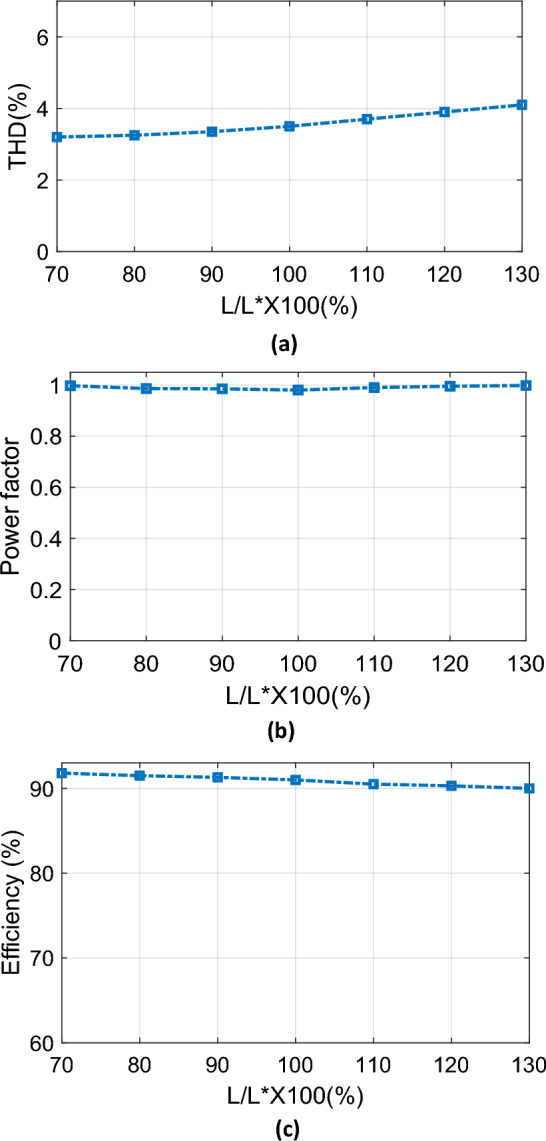
Measured characteristics of PFC system when the input inductance is changed: (**a**) THD of source current, (**b**) PF, (**c**) Efficiency.

**Figure 20:**
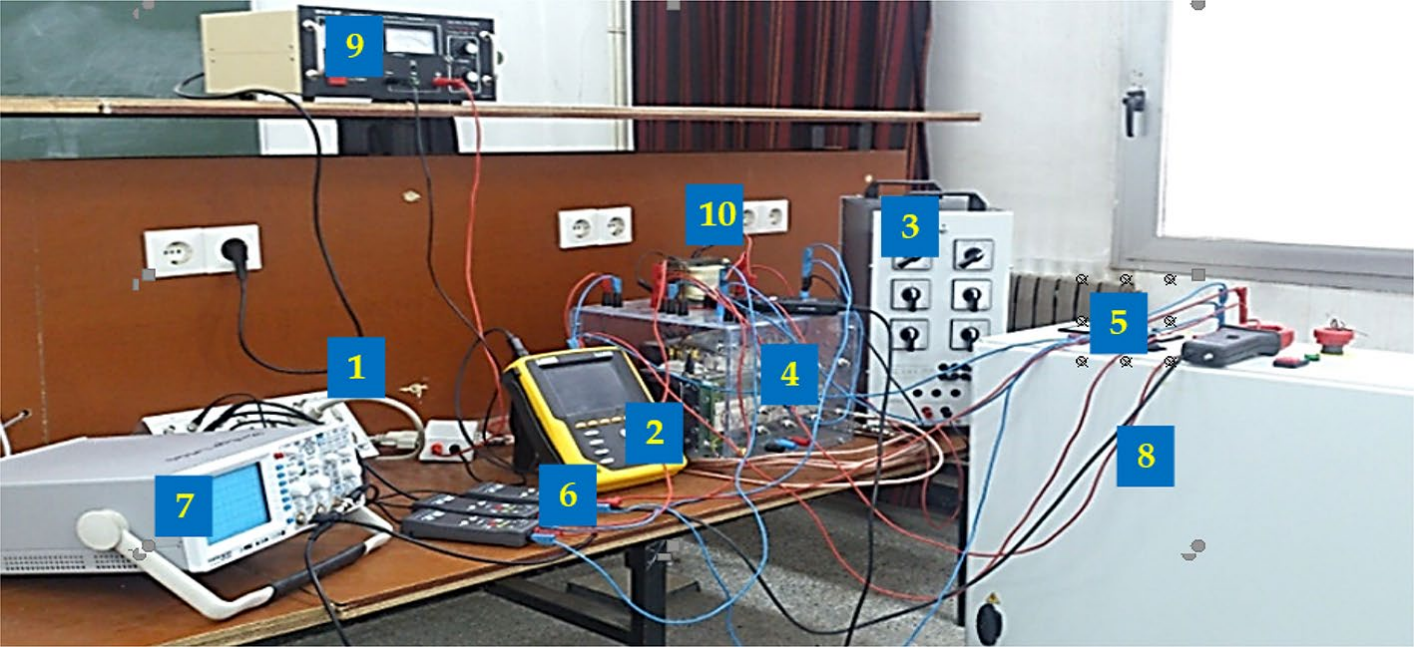
Experimental set up to test the suggested regulator; (**1**) dSPACE ds1104 card; (**2**) power analyzer; (**3**) output resistor; (**4**) SEMIKRON converter; (**5**) current sensor; (**6**) voltage sensor; (**7**) scope; (**8**) AC power supply, (**9**) DC power supply, (**10**) input inductor.

**Figure 21:**
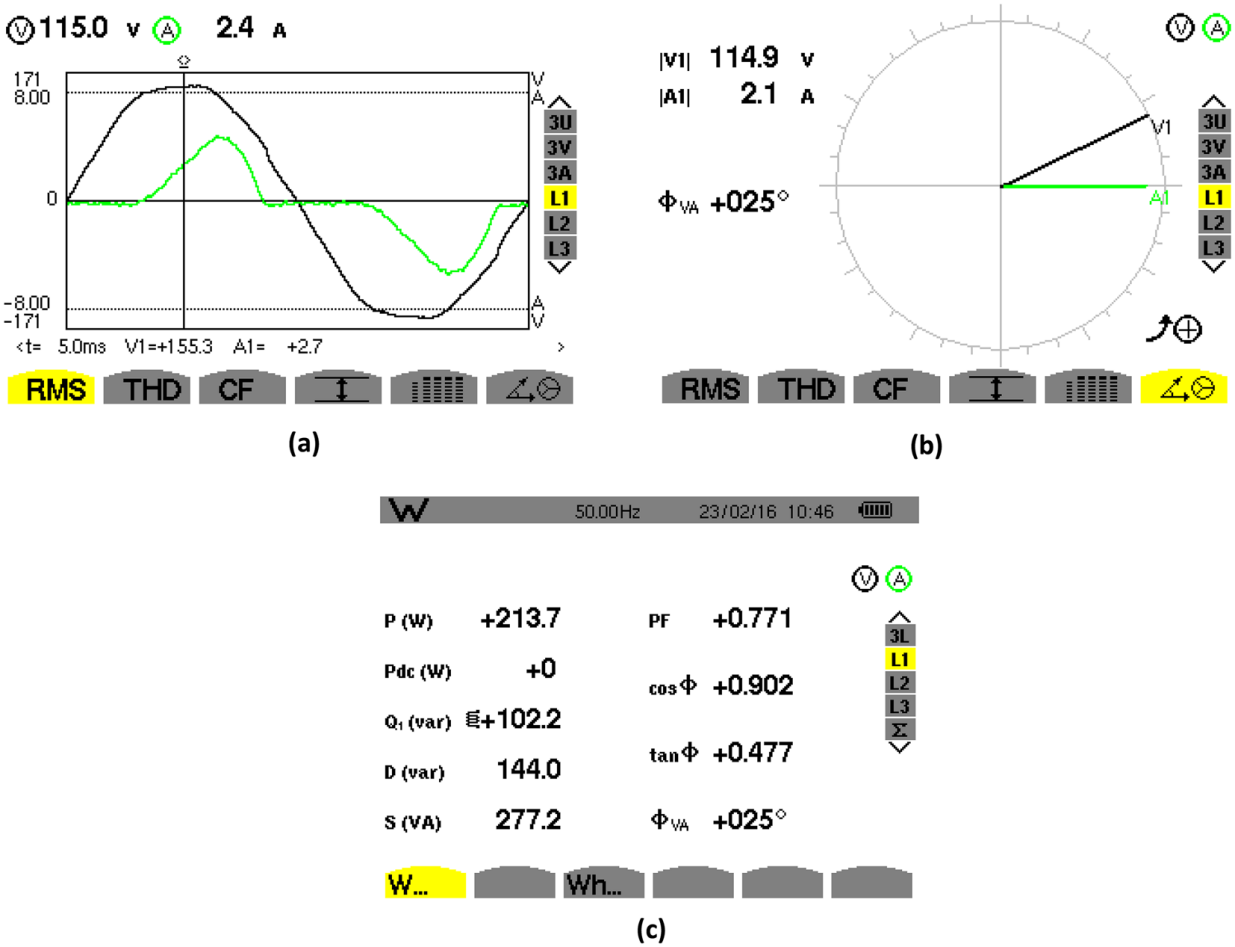
Measured results without PFC: (**a**) input voltage (Black) and source current (Green), (**b**) Fresnel diagram, and (**c**) PF.

The sensitivity of the suggested predictive controller to parameter changes was examined by varying the input inductor value and monitoring the performance of the proposed PFC system. Seven measured points are recorded through a specified range of variation from 70 to 130% of the real input inductor. Figures 19, 20, 21 depicts the characteristics of the source current THD, PF and efficiency when the input inductor is changed. The inductance values are 14 mH (70%), 16 mH (80%), 18 mH (90%), 20 mH (100%), 22 mH (110%), 24 mH (120%), and 26 mH (130%). It is desirable that these characteristics of the rated inductance can be maintained even when the inductance is changed. Therefore, input inductor variations have no significant effect on the source current THD, PF or efficiency. In addition, since the characteristics in Figs. 19, 20, 21 are almost non-variable, it can be confirmed that the PFC system is able to maintain the CCM operation despite the occurrence of variations in the input inductor when using the suggested predictive technique.

## Identified practical challenges

There are numerous practical challenges that can occur during the preparation and implementation of the proposed control technique. The following section outlines and elaborates on the practical challenges arising from the chosen proposed control technique:

1. The proposed control technique requires an appropriate model of the PFC system, which is then used for the computation of the control action. This model must be sufficiently precise, in order to yield valid predictions of the relevant variables, but at the same time, the model must be as simple as possible for the optimization task to be computationally tractable and numerically stable.
2. In the proposed control technique, all predictions begin from an initial state. The PFC model should be initialized to the measured/estimated current state. Depending on what the state of the PFC system is described, it might be impossible to measure everything directly. In this case, a Kalman filter can be used to estimate the current state of the PFC system and the estimate is used as initial/ current state for control.
3. A third challenge of proposed control technique is ensuring the stability and robustness of the closed-loop system in the presence of disturbances. Although proposed control technique can handle a certain degree of disturbance, it still suffers from performance degradation or instability if the disturbance is large or unpredictable. Therefore, we need to design the predictive current controller with stability and robustness considerations, such as using adaptive method or stochastic predictive control.
4. A final challenge of proposed control technique is dealing with the practical issues that arise when implementing a predictive controller on a real system. These issues include sensor noise, measurement delays, actuator limitations, communication failures, and software errors. These issues can affect data quality, the feasibility of the control action, and the reliability of the control loop. Therefore, we need to implement the proposed control technique with care and attention, such as using filtering, anti-windup, fault detection, and testing methods.

## Experimental validation

Experiments were carried out under CCM operation (output power of 500 W, input AC voltage of 70 Vrms) in LAS laboratory of the University of Setif-1 using a dSPACE ds1104 control board in order to confirm the simulation results of the considered PFC converter with ANFIS and predictive current controller. The power circuit of the proposed PFC system is mainly constituted by IGBT 1200 V, 50 A (SKM 50 GB 123D), a 1 − φ auto-transformer (0–400 Vrms), a 1 − φ diode bridge rectifier 1800V, 60 A (SKD62/18), and a DC-load. An external card based on IXDP630 chip and an isolated driver circuit with SKHI-22 were utilized to ensure the digital dead-time and the isolation of the PWM signal. The current and voltages of the PFC circuit were measured using a Fluke i30s current sensor and GW Instek GDP-050 voltage transducers. The THD and PF of the studied PFC system were computed using a Chauvin Arnoux C.A 8335 power analyzer and a digital oscilloscope (200MHz Mixed Signal CombiScope HM2008) ensuring the presentation of the experimental results. The experimental set up utilized in the laboratory is depicted in Fig. 19. The values of the PFC circuit parameters utilized for both experimental prototype and simulation studies are same as given in Table 3.

## Hardware results and discussion

The output DC voltage and output DC resistor were varied during experiments to test the robustness of the PFC system. In order to demonstrate the performance of the proposed method, four different experiments were analyzed. In the first experiment, the system is tested in the steady-state without PFC under constant output voltage and load (Sect. 6.1). In the second experiment, the PFC system is tested in the steady-state with PFC under constant output voltage and load (Sect. 6.2). In the third experiment, the tracking behavior of the suggested method is evaluated based on the variations of the output voltage reference (Sect. 6.3). In the fourth experiment (Sect. 6.4), the control behavior is investigated under sudden load variation by connecting or disconnecting the parallel load.

### Steady-state waveforms without PFC

Figures 20, 21 show the experimental steady-state waveforms without PFC system for constant-load and AC input voltage of 115Vrms. The AC input voltage in Fig. 20a is sine-shaped, but the source current is not, and has been shifted at a large angle with respect to the AC input voltage as can be seen in Fig. 20b, leading to a distortion condition. The PF between the source current and the AC input voltage has been determined as 0,771 as illustrated in Fig. 20c. The distortion state can be highlighted in Fig. 21b where it displays the harmonic spectrum of the source current.

### Steady-state waveforms with PFC

**Figure 22:**
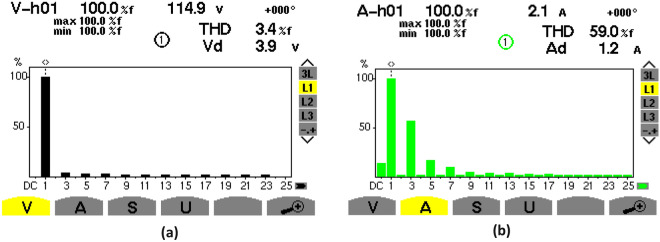
Harmonic spectrum without PFC: (**a**) THD of input voltage, (**b**) THD of source current.

**Figure 23:**
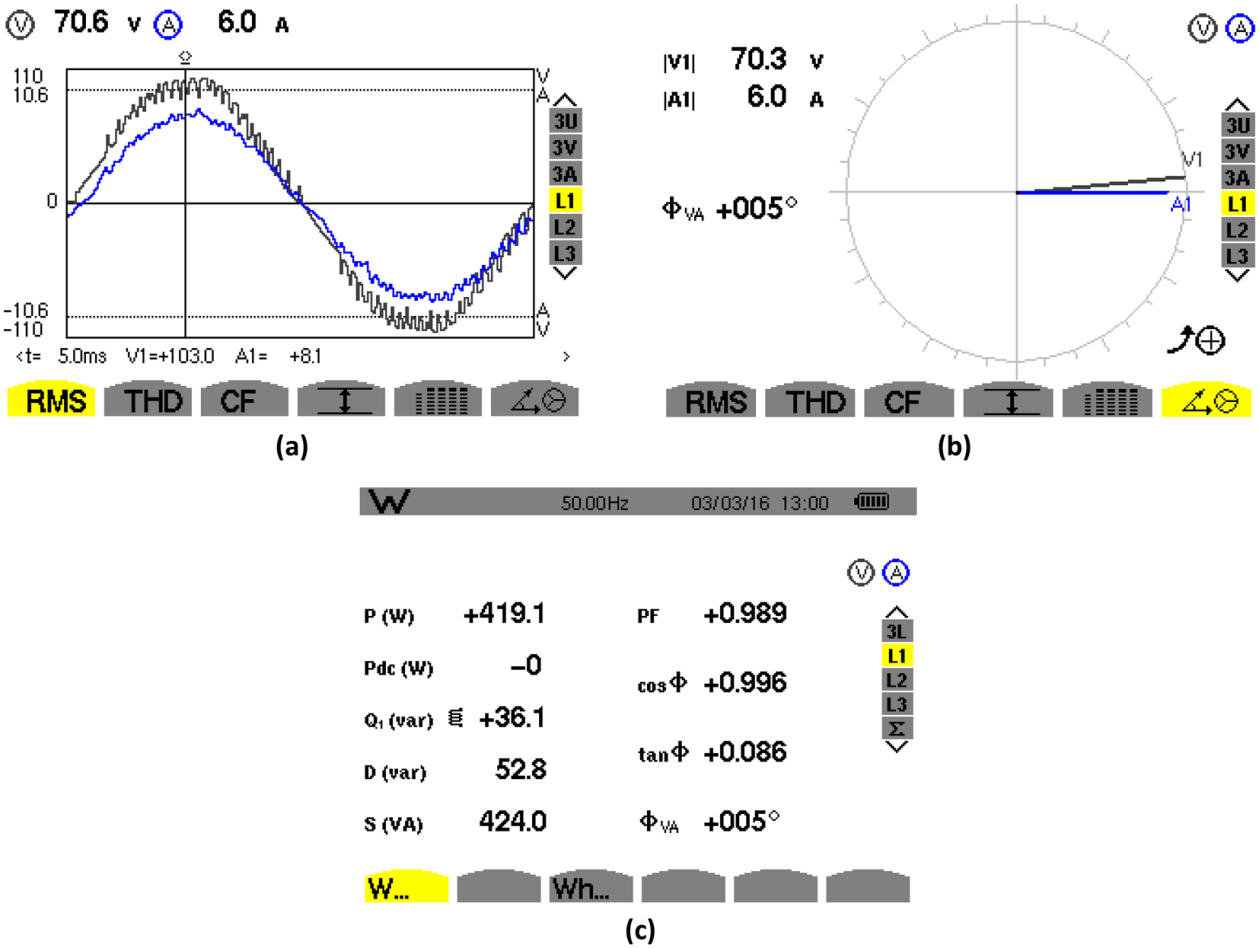
Measured results of: (**a**) input voltage (Belau) and source current (black), (**b**) Fresnel diagram, and (**c**) PF.

**Figure 24:**
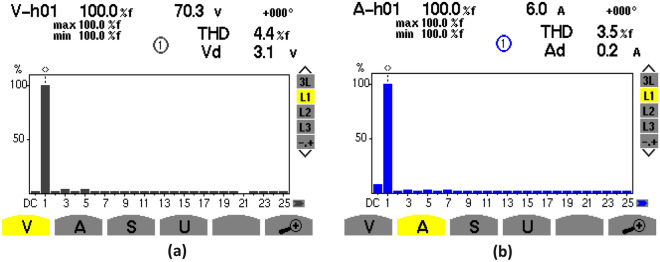
Harmonic spectrum of: (**a**) input voltage, (**b**) source current.

**Figure 25:**
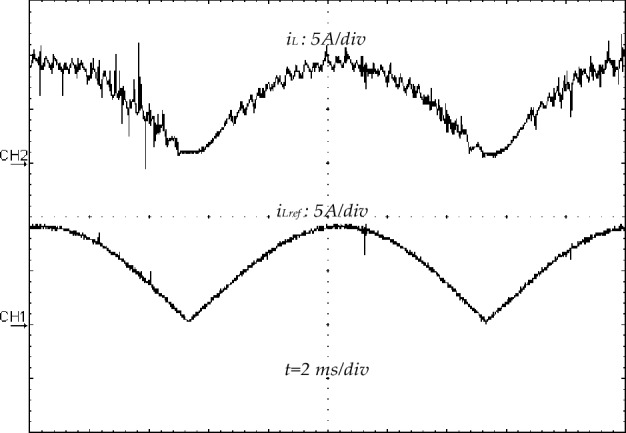
Inductor current (*i*_*L*_) (CH_1_) and its reference (*i*_*L_ref*_) (CH_2_) (5A/div).

The experimental steady-state waveforms with PFC under constant-load and DC output voltage of 250 V are depicted in Figs. 22, 23, 24, 25. It can be observed that the steady-state behavior is very satisfying where the source current (is) is in sinusoidal waveform (Fig. 22a) with low THD at AC mains (3.5%) (Fig. 21b) that complies with the IEEE-519 standard (THD ≤ 5%)^3^, and the power factor is about 0.990 (Fig. 22c), which enhances the energy efficiency via unity PF operation. Moreover, the measured inductor current has the same form of its reference as indicated in Figs. 23, 25 displays the steady-state waveforms of the input voltage and source current. A sinusoidal source current that is in phase with the input voltage has been obtained.

### Experimental results under output voltage variations

**Figure 26:**
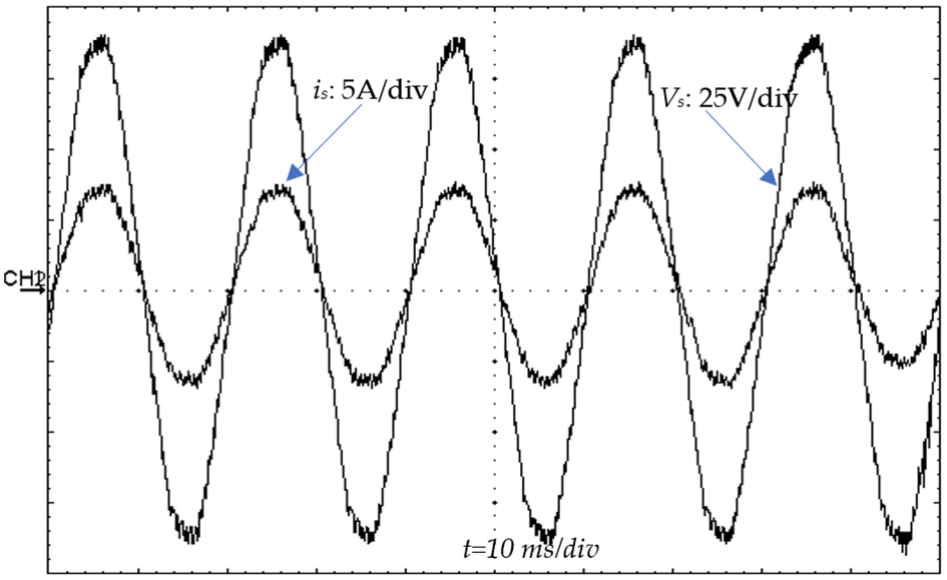
Source current (*i*_*s*_) (CH_1_) (5A/div) and input voltage (*v*_*s*_) (CH_2_) (25V/div).

During this experiment, the reference output voltage steps up from 180 V (100%) to 250 V (139%) and steps down from 250 to 180 V, respectively. The results for the con-trolled output voltage (*v*_*dc*_) as well as the input current (*i*_*s*_) are shown in Fig. 26. It can be seen from this figure that the controlled output voltage tracks their desired value (*v*_*ref*_) in a very satisfying manner without noticeable overshoot. The DC-link voltage reached its new reference (250 V) after 0.195 s and need 0.180 s to return to 180 V. Moreover, the input current is presented by its sinusoidal form, which leads to the functioning of our system under unitary PF.

### Variations experimental results under load variations

**Figure 27:**
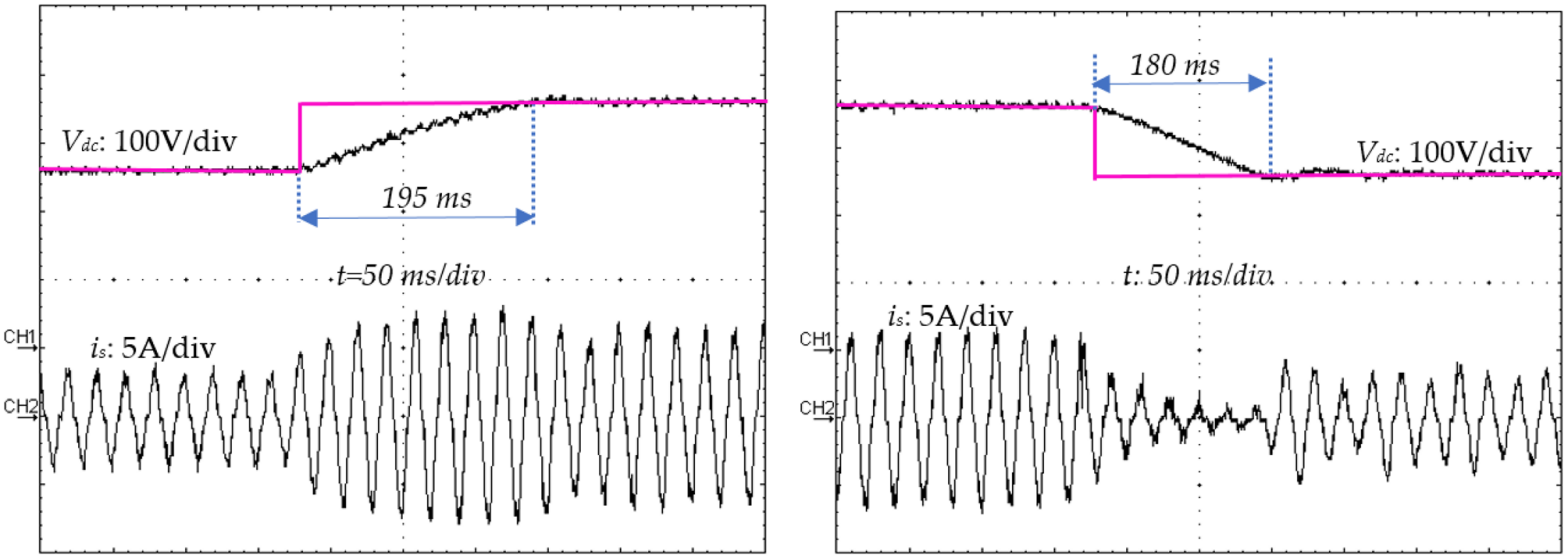
Output voltage variations (*v*_*ref*_) from 180 to 250 V and inversely.

**Figure 28:**
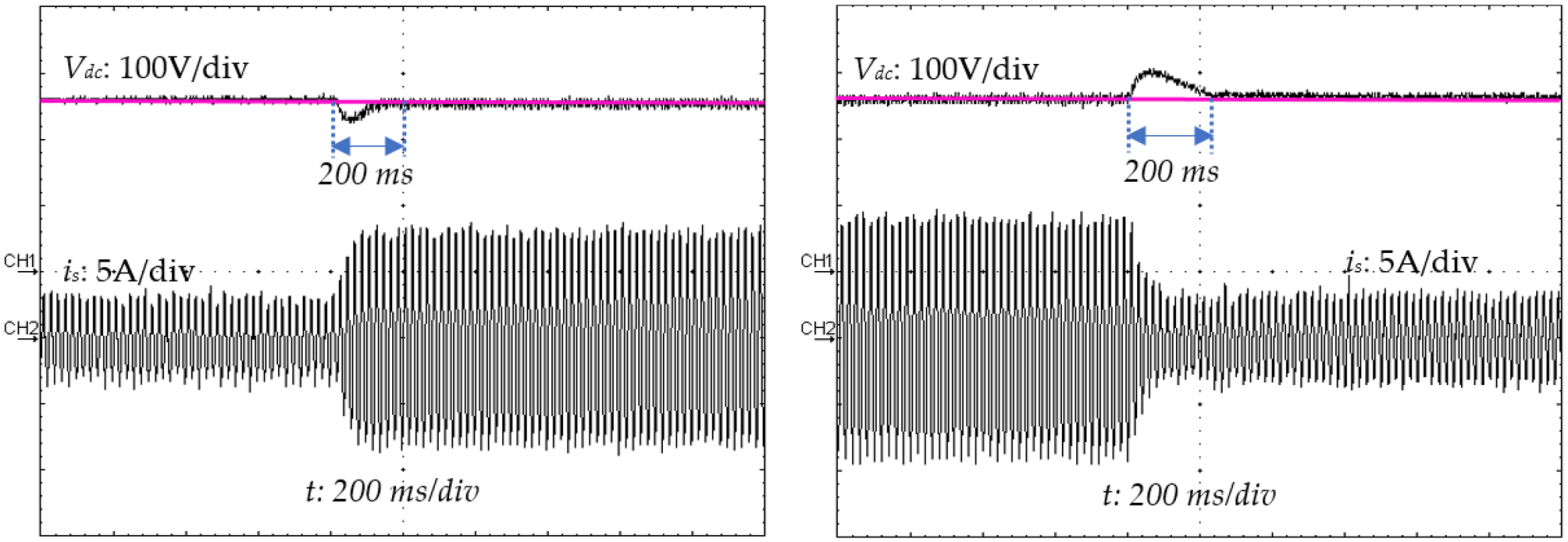
Output voltage (*v*_*dc*_), and source current (*i*_*s*_) transient responses during a DC-load change.

The proposed control strategy should also be robust enough to handle this kind of disturbance. Hence, the load is varied during this experiment from 100 Ω (full load) to 50 Ω (part load) and back to 100 Ω as shown in Fig. 27. The obtained experimental result displays an almost sinusoidal input current (*i*_*s*_) waveform and a constant output voltage (*v*_*dc*_), very close to its reference value (*v*_*ref*_) with presence of a slight transient drop and rise of 0.2 s (Fig. 28).

All the experimental results (the recovery time (*t*_*r*_), the response time (*t*_*s*_), and the output voltage overshoot/undershoot (*∆v*_*0*_)) of the suggested approach are summarized in Table 4 and compared to reference^15^ and reference^50^, with respect to the change in the load resistor (*R*) and the reference output voltage (*∆v*_*ref*_). According to the results given in Table 4, it is clearly visible that these results are satisfactory and competitive compared to those presented in references^15^ and^50^, respectively.

**Table 4:** Comparison of experimental results.

	Increasing load		Decreasing load		Increasing vref		Decreasing *v*_*ref*_	
	*∆v*_*0*_(*V*)	*t*_*r*_(*s*)	*∆v*_*0*_(*V*)	*t*_*r*_(*s*)	*∆*_*vref*_(*V*)	*t*_*s*_(*s*)	*∆v*_*ref*_(*V*)	*t*_*s*_(*s*)
Proposed method	18	0.2	20	0.2	70	0.19	70	0.18
Ref ^15^	25	0.18	25	0.15	40	0.1	40	0.1
Ref ^50^	14	0.4	13	0.3	38	0.5	38	0.5

After the previous figures, and Table 4, It is obvious that the proposed control approach based on ANFIS voltage controller and predictive current control is a good solution to the various problems encountered during the operation of the single-phase PFC boost converter device, thus ensuring significant energy saving.

## Conclusions and future research recommendations

This paper has described a novel control scheme to improve the power factor of AC/DC boost rectifier based on adaptive neural-fuzzy inference and predictive current controller where simulation and real-time implementation study is presented. Various experiments tests have been performed in different working conditions. Sudden load changes and reference output voltage variation were considered which are the typical industrial requirements. The efficiency of the proposed control approach has been proved through simulations and experiments. Obtained practical results are as follows:

- A steady-state analysis has shown that the power factor has reached the value of 0.990 and the THD of the input current is less than 5% that complies with the IEEE-519 standard;
- A transient state analysis has shown that the output voltage is unaffected during output load variations with negligees steady-state error;
- The time required to reach the desired value during variations in output voltage is very smaller. Indeed, to increase the measured output voltage to its reference, a time of 200 ms is sufficient to achieve this objective.

Through these practical results, the ANFIS controller has proven its effectiveness with re-gard to the monitoring of imposed references and the rejection of disturbances affecting the studied system. In addition, the combination of this intelligent controller with a predictive strategy has significantly improved the power quality of the power grid.

Further exploration of advanced control strategies, such as model predictive control (MPC) or fault-tolerant operation, is recommended to enhance the performance and reliability of AC/DC boost rectifiers. Investigating multi-objective optimization techniques could aid in developing control schemes that effectively balance power factor correction, efficiency, and transient response characteristics. Additionally, conducting hardware-in-the-loop (HIL) experiments and real-time hardware implementations would provide valuable insights into the practical applicability of proposed control algorithms. Furthermore, studying the integration of AC/DC boost rectifiers into smart grid environments and analyzing their role in supporting grid stability and facilitating renewable energy integration would be crucial for future research endeavors.

## Data Availability

The datasets used and/or analysed during the current study available from the corresponding author on reasonable request.
